# Protocol to assess rewarding brain stimulation as a learning and memory modulating treatment: Comparison between self-administration and experimenter-administration

**DOI:** 10.3389/fnbeh.2022.1046259

**Published:** 2022-12-15

**Authors:** Laia Vila-Solés, Soleil García-Brito, Laura Aldavert-Vera, Elisabet Kádár, Gemma Huguet, Ignacio Morgado-Bernal, Pilar Segura-Torres

**Affiliations:** ^1^Departament de Psicobiologia i de Metodologia de les Ciències de la Salut, Institut de Neurociències, Universitat Autònoma de Barcelona, Barcelona, Spain; ^2^Departament de Biologia, Universitat de Girona, Girona, Spain

**Keywords:** intracranial self-stimulation, medial forebrain bundle (MFB), brain reward system, learning, memory

## Abstract

Intracranial electrical self-stimulation (ICSS) is a useful procedure in animal research. This form of administration ensures that areas of the brain reward system (BRS) are being functionally activated, since the animals must perform an operant response to self-administer an electrical stimulus. Rewarding post-training ICSS of the medial forebrain bundle (MFB), an important system of the BRS, has been shown to consistently improve rats’ acquisition and retention in several learning tasks. In the clinical setting, deep brain stimulation (DBS) of different targets is currently being used to palliate the memory impairment that occurs in some neurodegenerative diseases. However, the stimulation of the MFB has only been used to treat emotional alterations, not memory disorders. Since DBS stimulation treatments in humans are exclusively administered by external sources, studies comparing the efficacy of that form of application to a self-administered stimulation are key to the translationality of ICSS. This protocol compares self-administered (ICSS) and experimenter-administered (EAS) stimulation of the MFB on the spatial Morris Water Maze task (MWM). c-Fos immunohistochemistry procedure was carried out to evaluate neural activation after retention. Results show that the stimulation of the MFB improves the MWM task regardless of the form of administration, although some differences in c-Fos expression were found. Present results suggest that MFB-ICSS is a valid animal model to study the effects of MFB electrical stimulation on memory, which could guide clinical applications of DBS. The present protocol is a useful guide for establishing ICSS behavior in rats, which could be used as a learning and memory-modulating treatment.

## Graphical Abstract

Comprehensive timeline of an experiment using self-administered or experimenter-administered intracranial stimulation to modulate learning and memory. 
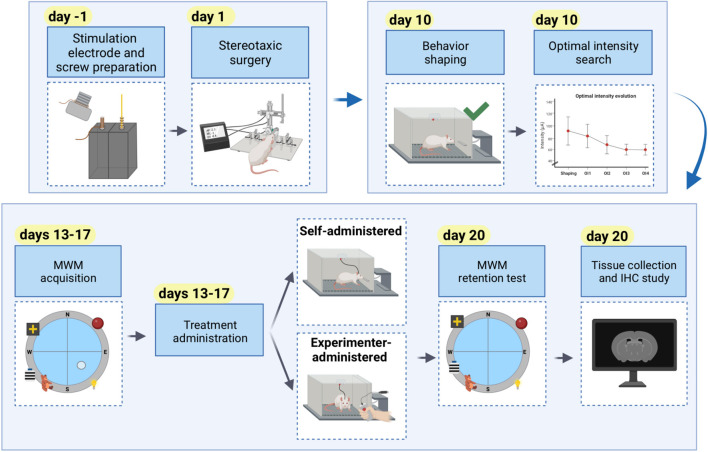

## Introduction

Motivational and reinforcement processes are directly involved in operant conditioning (Bamford et al., 2018). However, data from many animal studies have demonstrated that the stimulation of regions belonging to the brain reward systems (BRS) also modulates learning beyond operant conditioning, by acting as a facilitating treatment for different learning and memory tasks (Morgado-Bernal and Segura-Torres, 2022). One of the main regions of the BRS, and one of the most commonly targeted is the medial forebrain bundle (MFB) in the lateral hypothalamus (Milner, 1991). Animals implanted with electrodes to the MFB self-administer the stimulation trains, due to the reinforcing nature of the stimulation. This kind of administration paradigm is known as intracranial self-stimulation (ICSS), formerly described by Olds and Milner (1954).

Medial forebrain bundle-intracranial electrical self-stimulation, especially when administered post-training, is capable of improving the consolidation of both non-declarative (Aldavert-Vera et al., 2013; García-Brito et al., 2017) and declarative memory (Chamorro-López et al., 2015; García-Brito et al., 2020), and even promoting the functional recovery of induced memory deficits (Segura-Torres et al., 2010; Kádár et al., 2014). ICSS of the MFB also manages to activate plasticity processes in different circuits involved in several types of memory (Aldavert-Vera et al., 2013; Kádár et al., 2014; Huguet et al., 2020; Puig-Parnau et al., 2020). Despite the promising results in animal models, clinical trials using deep brain stimulation (DBS) to alleviate memory deficits in disorders such as Alzheimer’s disease usually target discrete memory-related regions (i.e., fornix, entorhinal cortex, or Basal nucleus of Meynert) (Lyons, 2011; Aldehri et al., 2018), rather than the MFB or any other BRS regions. On the other hand, the MFB is currently being used as a therapeutic target in disorders such as anhedonia and depression (Bewernick et al., 2017; Fenoy et al., 2018). Taken all together, these results suggest that targeting MFB fibers could be useful in the treatment of diseases presenting diverse symptomatology, including emotional, motivational and memory alterations, which could be present in Alzheimer’s disease (Bennett and Thomas, 2014; Wei et al., 2020).

While ICSS is a procedure in which animals self-administer the electrical stimulation, DBS in humans is not self-administered. Thus, in order for animal research to inform future clinical studies, it is important to determine whether the desired therapeutic effects can be achieved by either form of administration.

The primary goal of this protocol is to compare self-administered (ICSS) and experimenter-administered (EAS) stimulation of the MFB in terms of its effectiveness in enhancing spatial memory consolidation. In order to do this, the animals are trained in a hippocampus-dependent spatial task in the Morris Water Maze (MWM) (Vorhees and Williams, 2014b), and receive a post-training treatment (ICSS or EAS) immediately after each of the 5 MWM acquisition sessions. The effects of the stimulation of the MFB are evaluated every 24 h in the acquisition sessions, and in the retention test 72 h after the last acquisition session. In addition, the neural activation in different brain regions was evaluated 90 min after retention, using a c-Fos immunohistochemistry protocol.

The secondary goal of this protocol is to serve as a comprehensive how-to guide in the establishment of consistent ICSS behavior in rats, in order to use it as a treatment to study its relationship to learning and memory. We provide detailed explanations for the preparation of homemade electrodes, electrode implantation surgery, and procedures to obtain self-administration behavior. Moreover, we specify the parameters for the use of two forms of rewarding stimulation administration (ICSS and EAS) as a treatment to improve performance in a spatial task in the MWM. Although this protocol describes the post-training administration of the treatment, some aspects could also be applied to the design of experiments aimed at assessing the effects of MFB stimulation in other phases of learning and memory. The procedure and outcome for the learning and memory task in the MWM as well as for the c-Fos expression immunochemistry are also included in the protocol.

Moreover, the specific steps to establish ICSS behavior safely and effectively may also be of interest to researchers working in fields of study in which reward is involved, such as addictive behaviors (Baird et al., 2021). Carlezon and Chartoff (2007) provide a more detailed description of the procedure to follow in order to assess motivational states, using ICSS behavior as a dependent variable.

Given that basic research using animals can sometimes bring about unexpected or even potentially harmful consequences, it is of paramount importance to establish well-characterized protocols in order to obtain reliable results with minimum impact to the animals’ welfare. On that basis, a comprehensive list of the most frequent issues and troubleshooting is provided. All procedures were carried out in compliance with the Directive 2010/63/EU and were approved by the institutional animal care committee.

## Materials and equipment

### Stimulation electrode and ground screw materials

**FIGURE 1:**
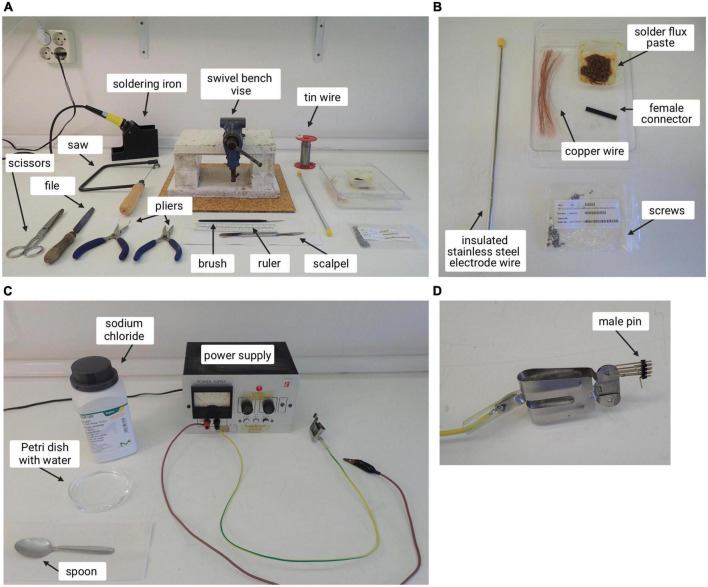
Setup of the working area with instruments needed to build electrodes and ground screws. **(A)** Materials and tools used for the preparation of stimulation electrodes and ground screws. **(B)** Magnification of some of the materials needed for this process. **(C)** Power supply and materials used to check electrode current. **(D)** Male pin used to attach the electrode prepared to the power supply.

Materials shown in Figure 1.

a. General instruments (pliers, file, ruler, scissors)
b. Scalpel
c. Saw
d. Soldering iron
e. Swivel bench vise
f. Insulated stainless steel electrode wire
g. Female connector
h. Screws
i. Brush and solder flux paste
j. Tin wire
k. Copper wire
l. Power supply
m. Petri dish with water
n. Sodium chloride
o. Spoon
p. Male pin

### Surgery equipment

**FIGURE 2:**
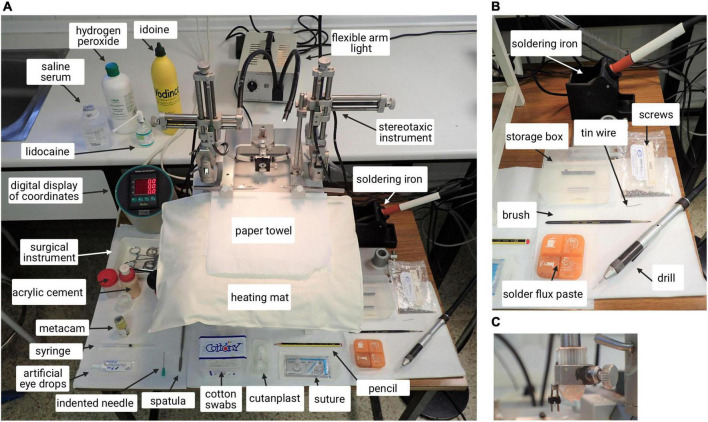
Picture of equipment and surgery area. **(A)** Stereotaxic instrument with labeled materials. **(B)** Instruments and materials. **(C)** Magnification of the male pin attached to the stereotaxic arm. The female connector of the electrodes fabricated previously is attached to this male pin for support.

Surgery equipment shown in Figure 2.

a. Paper towel
b. Hair trimmer
c. Artificial eye drops
d. Brush
e. Heating pad
f. Surgical lights with flexible arms
g. Digital display of coordinates
h. Gas extractor
i. Surgical instrument (scalpel, spatula, x4 curved mosquito forceps, scissors, x1 straight mosquito forceps, standard forceps)
j. Lidocaine
k. Iodine
l. Meloxicam
m. Hydrogen peroxide
n. Cotton swabs
o. Pencil
p. Sterile 0.9% saline solution
q. Surgical drill
r. Electrodes (previously prepared)
s. Screws and ground screws (previously prepared)
t. Acrylic cement (mixture)
u. Suture materials
v. 21G needle, indented tip
w. Sterile-absorbable hemostatic gelatin (Cutanplast^®^)

### Electrical brain stimulation equipment

#### Skinner box

**FIGURE 3:**
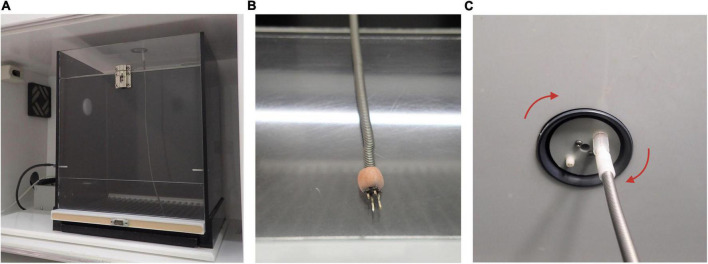
Picture of Skinner box used and details. **(A)** Clear door Skinner box used for intracranial electrical self-stimulation (ICSS)/experimenter-administered stimulation (EAS) administration. Note that there is not a high level of lighting. This is due to standardized behavioral testing conditions from our lab. **(B)** Male pin is used to connect the stimulator to the female entrance of the stimulation electrode. Note that acrylic cement is used to fix the male pin to the cable connected to the stimulator. **(C)** The wire connected to animals’ female pin is attached to the electrode swivel located in the Skinner box ceiling order to provide rotational movement to animals.

For all experimental conditions (ICSS, EAS, or non-stimulated animals), a conventional 25 × 20 × 20 cm Skinner box with a lever inserted in one of the walls was used, as shown in Figure 3.

#### Stimulator

**FIGURE 4:**
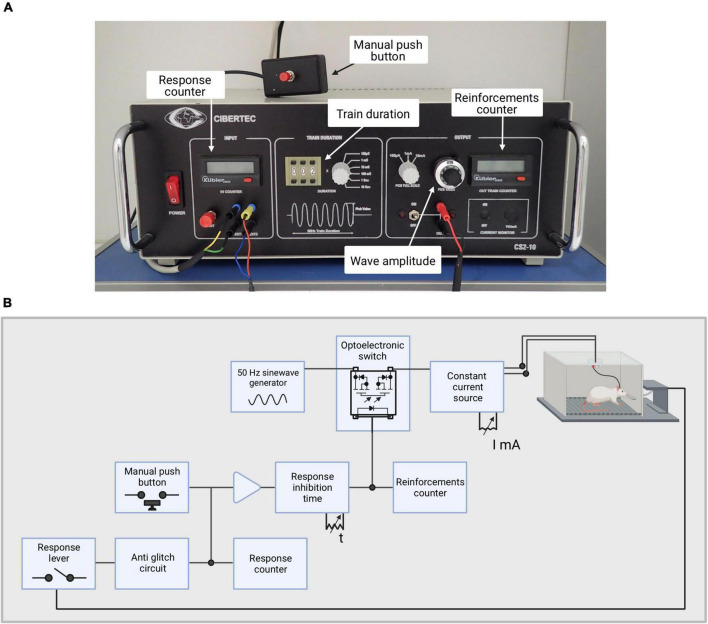
Stimulator used for this experiment. **(A)** Picture of the stimulator with its main features depicted. **(B)** Illustration depicting the stimulator circuit.

The stimulator used for these experiments is the model CS2-10 (previously the model CS20 IM) from Cibertec, which creates trains of bipolar sinusoidal waves at a constant current at 50 Hz (see Figure 4B). The output stimulation circuit is isolated optoelectronically. The duration of the trains can be adjusted (in this case we use 0.3 ms). The triggering of the train can be performed manually by a button or a response lever inside the Skinner box. The triggering signal is applied into an anti-rebound circuit that prevents unwanted stimulations. Before reaching the stimulator, the triggered signal goes through a circuit of pulse inhibition, which can be adjusted to different times. The goal of this system is to not let another signal go through until the preset inhibition time has passed. The stimulator has two digital displays, the Responses, in which all responses are marked, and the Reinforcements, in which all stimuli received by the animal are shown, as shown in Figure 4A.

### Behavioral apparatus

**FIGURE 5:**
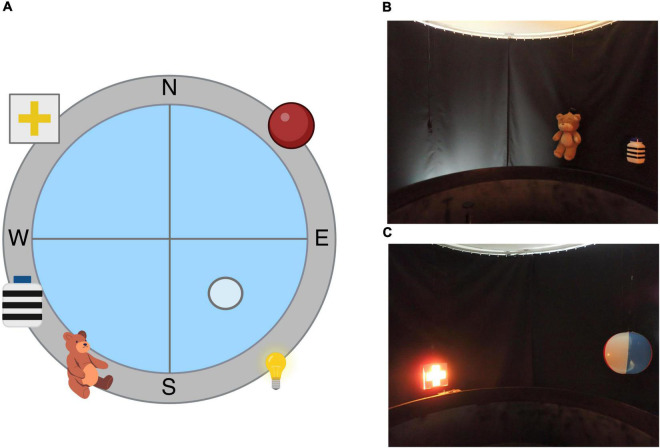
Morris Water Maze apparatus. **(A)** Illustration depicting the spatial Morris Water Maze configuration used in our lab. **(B)** Picture of the objects used in the South-east (SE) and South-west (SW) quadrants. **(C)** Picture of the objects used in the North-west (NW) and North-east (NE) quadrants.

The behavioral apparatus used in this experiment to test spatial learning and memory was the Morris Water Maze (MWM). The MWM is a maze in which subjects have to find a submerged platform in the middle of the four quadrants of the pool. The tank is virtually divided into four quadrants, defining four cardinal points: North (N), South (S), East (E), and West (W), as observed in Figure 5A. Subjects have to identify and use the spatial cues hung around the pool in order to find the platform and escape the pool. The cues used in this experiment are; North-east (NE): beach ball with different color stripes, South-east (SE): indirect light of a lamp in the curtain, South-west (SW): teddy bear and a white plastic cylinder with horizontal black stripes, and North-west (NW): cross cutout in a box with a light inside, as observed in Figures 5B,C.

Swim paths were recorded using a closed-circuit video camera (Smart Video Tracking System, Version 2.5, Panlab) with a wide-angle lens 1.75 m above the center of the pool.

See Table 1 for all reagents and resources with their source and identifiers.

**TABLE 1:** Description of all reagents (subjects, chemicals, solution preparations, other relevant tools, and software) used in this protocol with their corresponding source and identifiers.

Reagent or resource	Source	Identifier
**Subjects**		
Wistar rats	Charles River www.criver.com	In this experiment, the offspring of the animals bought were used to avoid stress effects due to transportation.
**Chemicals**		
Solder flux paste	Shoptronica www.shoptronica.com	Ref. 0689593987280
Sodium chloride	Merck, Sigma Aldrich www.sigmaaldrich.com	Ref. 1064040500
Artificial eye drops	Centauro www.centauro.es	Lubrithal 10gr Ref. 5701170313291
Lidocaine	Royal Dent https://www.royal-dent.com/	Ref. 12408
Iodine	Centauro www.centauro.es	Ref. 8413204701031
Metacam	Centauro www.centauro.es	Ref. 4028691555438
Hydrogen peroxide	Centauro www.centauro.es	Ref. 8410088000144
Intravenous serum	Centauro www.centauro.es	Ref. 4030539076609
Acrylic cement	Proclinic www.proclinic.es	Solid resin (1 kg): Ref. AVCPP0801000 Liquid resin (1000 ml): Ref. AVCPV01000
Isoflurane	Centauro www.centauro.es	Ref. 8436025713298
TRIS	Serva www.serva.de/enDE	Ref. 37190.02
Sodium chloride	PanReac AppliChem www.itwreagents.com/	Ref. 131659.1210
Bovine Serum Albumin (BSA)	EMD Millipore Corp. www.merckmillipore.com/	Ref. 12650-250GM
Triton X-100	Merck Group www.sigmaaldrich.com	Ref. X100-500 ml
Mouse anticFos primary antibody	Santa Cruz Biotechnology www.scbt.com	Ref. 166940
Biotinylated goat anti-mouse-IgG secondary antibody	Jackson Immunoresearch www.jacksonimmuno.com	Ref. 115-065-166
Streptavidin–HRP	Perkin Elmer https://www.perkinelmer.com/es/	Ref. NEL750001EA
3,3’-Diaminabenzidine (DAB)	Vector Laboratories www.vectorlabs.com	Ref. SK-4100
Pertex mounting medium	Medite GmbH www.medite.de/en/	Ref. 41-4011-00
**Solution preparations**		
**Tris-Buffered Saline (TBS) 10× ph.7.6 (Stock solution)**		
60.6 gr TRIS Serva + 88 gr. NaCl in 1 L Milli-Q water		
**Other**		
Scalpel blade	Centauro www.centauro.es	Ref. 1110910V
200 μm diameter insulated stainless steel electrode wire–straightened for 30 cm	Bilaney www.bilaney.com	Ref. 008SW/30S
Female connector for electrode base	Diotronic S.A. www.diotronic.com	Ref. SSQ36SSTG
Male pins 2.54 mm	Diotronic S.A. www.diotronic.com	Ref. SSQ36SSTG
Screws	Harvard Apparatus www.harvardapparatus.com	Ref. CMA7431021
Tin wire	Diotronic S.A. www.diotronic.com	Ref. HIF00651
Copper wire	Diotronic S.A. www.diotronic.com	Ref. WIK01N
Power supply (20V/0.2A)	Discontinued	Model SK112
Stereotaxic equipment	Cibertec www.cibertec.es	https://www.cibertec.es/es/catalogo-de-productos/bydiscipline/88
Sterile-absorbable hemostatic gelatin (Cutanplast)	Uppermat www.uppermat.com	Ref. CU101024
Anesthesia equipment	Cibertec www.cibertec.es	https://www.cibertec.es/es/catalogo-de-productos/bydiscipline/37
Skinner box	Cibertec www.cibertec.es	https://www.cibertec.es/es/catalogo-de-productos/bydiscipline/91
Stimulator model CS2-10	Cibertec www.cibertec.es	Made to experiment specification.
**Software**		
Smart Video Tracking System, Version 2.5	Panlab–Harvard Apparatus	www.panlab.com
Image-J 1.43	Image-J	https://imagej.nih.gov/ij/
BioRender	BioRender	www.biorender.com

## Methods

### Stimulation electrode and ground screw preparation

Timing: 2 h (2 h/6 electrodes and 6 ground screws).

**Important considerations:** Always prepare the electrodes between 1 and 3 days prior surgery. This time period should be taken into account to prevent oxidation of the stimulation electrode. Gloves may complicate most steps of this procedure; instead, wash your hands carefully before starting and after finishing.

**FIGURE 6:**
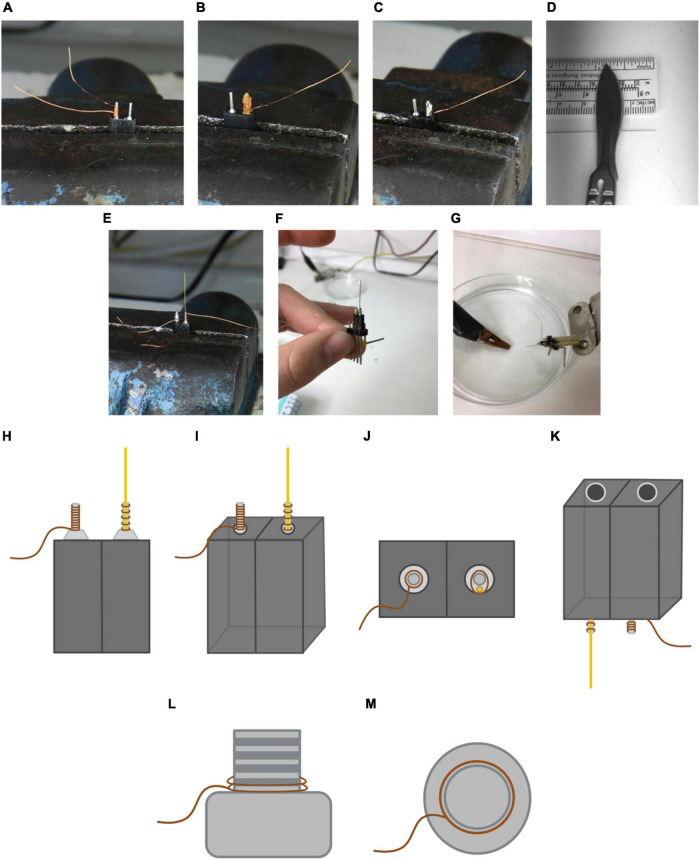
Preparation and end result of the stimulation electrode. **(A)** Placement of the copper wire around the first horn, covering it until the top. **(B)** Placement of the solder flux paste around the wire to optimize the soldering process. Note that in this picture the excess wire in the bottom part is already cut. **(C)** End result of the first horn once it is soldered. **(D)** 1.3 mm electrode bit cut. **(E)** Placement of the electrode wire on the second horn. Note that once one of the extremes is stripped of insulating material, this end must face downward when placing it on the horn. **(F)** Placement of the resulting device in a male pin to check its connectivity. **(G)** Current verification of the electrode wire and copper wire with a power supply and a solution of water with dissolved NaCl. **(H)** Frontal view of the resulting stimulation electrode. The brown lines represent the copper wire while the gold line represents the electrode wire. Note that the soldering tin is not shown. Not to scale. **(I)** Frontal-lateral view of the stimulation electrode. **(J)** Top view of the stimulation electrode. **(K)** Bottom-lateral view of the stimulation electrode. **(L)** End result of the ground screw. Lateral view of the ground screw. The brown line represents the copper wire. **(M)** End result of the ground screw. Top view of the ground screw. Not to scale.


1. Prepare all material necessary for this procedure in advance. Turn on the soldering iron.
2. Prepare 2-bases section using a saw. After sawing the sections, file any imperfections. Caution: Carefully check if the sawing has exposed any part of the metallic horns inside the base. If so, discard and obtain a new section.
3. Place the base in the swivel bench vise and secure it. Cut 7 cm of copper wire. Leave about 4 cm of wire to pull at the base and roll up the rest of the wire around the horn until it is completely covered. Leave around 1 cm excess wire at either end of the horn and cut the wire excess at the opposite end. Use the brush to collect some solder flux paste and cover the horn with it. Take the soldering iron, take a drop of tin and cover the horn (see Figures 6A–C). Caution: both the flux paste and the tin should always be applied from the top to bottom. If done in the opposite direction, the copper wire could be dislodged.
4. Cut another 7 cm length of copper wire. Place the center of it behind the other horn and roll it around the horn once to create a stable base.
5. Cut a 1.3 cm insulated electrode wire (Figure 6D). Once it is cut, scratch off 3–4 mm of one of the extremes using a scalpel in order to strip the insulating layer. Check with a magnifying glass if all the insulation has been correctly removed.
6. Place the stripped end of the electrode wire on the base made by the rolled wire. While holding the electrode, take one of the extremes of the wire and roll it around the electrode wire and the horn of the base 3–4 times in order to secure the electrode to the horn (see Figure 6E). Once it is attached, cut the excess copper wire at both ends and solder them. Important: the insulated end of the electrode wire should end up facing us, as shown in Figures 6H–K.
7. Once we have finished preparing our stimulation electrode, it must be functionally checked. Connect the base to a power supply attachment piece, as shown in Figure 6F. First, attach the power supply to the part of the base where the electrode wire is located. Introduce the tip of the electrode wire into the solution of salted water (avoid the copper wire touching the water), as shown in Figure 6G. If electrical current is running through the stimulation electrode, you should be able to see bubbles around the tip of the electrode wire and the power supply should indicate an increase in current levels. Repeat the same process for the copper wire (ground).
8. Check if the remaining length of the electrode wire is 1 cm long.
9. Store the stimulation electrodes in a sealed box until use.
10. To prepare the ground screw, place the screw in the swivel bench vise with its head facing downward and secure it.
11. Cut a 7 cm copper wire. Leave between 1 and 2 cm of copper wire and use the rest of it to roll it around the wire of the screw twice.
12. Cut the top extreme.

13. Use the brush to collect some solder flux paste and cover the copper wire.
14. Take a drop of tin with the help of the soldering iron and cover the copper wire. It is important to check if the copper wire ends up fully stuck. If the copper wire can still be moved around the screw, repeat this process until it is fully attached to the screw.
15. Once ground screws are prepared, keep them in a sealed box until used (see Figures 6L,M for the final result of the ground screw).

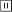
**Pause point**: No more than 4 days should pass between electrode and ground screw preparation and usage.

Check Desai et al. (2014) for other ways of solving rotational movement issues.

### Stereotaxic surgery

Timing: 1 day (30 min/rat).

The purpose of this step is to unilaterally implant a chronic electrode in the Medial Forebrain Bundle at the Lateral Hypothalamus. Stereotaxic coordinates in relation to Bregma are: –2.3 mm anterior-posterior, 2 mm medial-lateral, and 8.8 mm dorsal-ventral, according to the rat brain atlas (Paxinos and Watson, 2007). All experiments below are repeated for each rat. For our experiments, we use 3-months-old male Wistar rats from our breeding stock.

**FIGURE 7:**
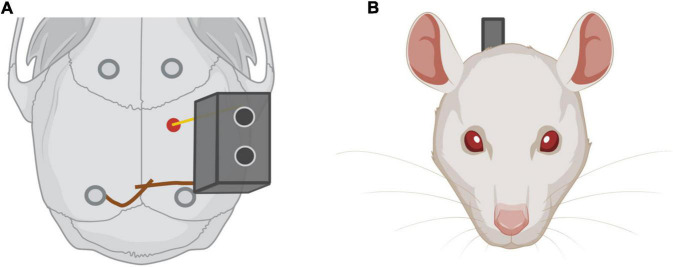
**(A)** Electrode implantation. In red, electrode implantation position in the rat skull. Note in dark gray the four screws implanted in the skull to improve the adhesion of the implant. The ground screw is implanted in the contralateral hemisphere regarding the electrode and near Lambda. **(B)** Rat frontal view with the electrode implanted.


1. Turn on a heating pad and place it in the surgery area. Cover the working space with paper. Turn on the lights and the digital marker.
2. Induce anesthesia placing the animal in the anesthesia induction chamber with 5% isoflurane for 3–4 min. Check breathing modifications to identify when the animal is fully anesthetized. Afterward, remove the animal from the chamber and shave the scalp fur with a trimmer, and put artificial drops over the eyes.
3. Place the animal in the stereotaxic apparatus placing the bar inside the ear canal. Check if the head is immobile and symmetry between both ears and eyes. Place the animal’s upper teeth in the nose clamp and adjust the gas mask around its nose.
4. Reduce the isoflurane rate to 2.5%. Note that each animal may need different concentrations to maintain anesthesia. Breathing frequency should be checked during the whole procedure in order to assess the adequacy of the anesthesia.
5. Administer lidocaine on the scalp and iodine with help of a cotton swab.
6. Administer 2 mg/kg of analgesic (Meloxicam) subcutaneously.
7. Take the scalpel and make an anteroposterior 1.5–2 cm incision. It is important to avoid going over the incision with new cuts. Instead, a clear incision should be done at once.
8. Use a spatula to remove the periosteum membrane toward the laterals and expose the skull.
9. Use four mosquito forceps to set aside the tissue and expose the skull, making sure the lateral sutures, as well as Bregma and Lambda are visible and accessible.
10. Clean the surface of the skull with hydrogen peroxide.
11. Place the electrode in the lateral arm. Check that the electrode is straight and that the tip is not bent.
12. Place the tip of the electrode on the edge of one of the lateral cranial sutures. Mark zero at the horizontal axis (Y) in the digital marker. Place the tip to the opposite lateral suture. Divide the number obtained in two and place the tip of the electrode in that coordinate.
13. Place the tip of the electrode on Bregma and mark zero in all coordinates of the digital marker (X, Y, Z).
14. Find Lambda and check the dorsoventral coordinate. If the difference is higher than 0.3 from Bregma, the inclination of the head should be readjusted.
15. Find the coordinates AP –2.3, ML –2 and mark them on the skull using a pencil. Remove the arm from the working area to avoid bending the electrode tip.
16. Apply two saline drops to the skull and drill a hole in the coordinate marked before.
17. Make two other holes anterior to the frontal sutures and two other holes anterior to posterior sutures (see Figure 7A).
18. Attach the screws to the skull. Important! The ground screw should be attached in the hole located in the contralateral hemisphere for the electrode and anterior to Lambda.
19. Break the meninges in the electrode hole using the bent tip of the 21G needle and introduce the electrode in the hole until –8.8 mm DV. In case of excessive bleeding, use Cutanplast^®^ to press down on the area until bleeding stops.
20. Use the soldering iron and tin wire to solder the copper wires from the electrode base and the ground screw together. Cut any excess wire (see Figure 7A).
21. Use the solid resin and the liquid resin to create a honey-like consistency cement. Slowly let it drip over the skull, making sure all the screws and half the base of the electrode are covered. The cement is dry when tapping it with a spatula produces a slightly metallic sound. Caution! As it dries, the cement should be carefully molded using a spatula to avoid the formation of sharp edges that could damage the skin. Do not push the electrode once it has been implanted and before the cement cures.
22. Perform one anterior and one posterior suture from the electrode implant. If there is a part of the skull exposed, perform more sutures where needed. The base of the electrode should be exposed, as shown in Figure 7B.
23. Clean the wound with iodine using a cotton swab.
24. Withdraw the animal from the stereotaxic apparatus and place it in its homecage. The homecage should remain over a heating mat until the animal awakes. Place some pellets inside the cage to facilitate food intake during the following hours.

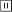
**Pause point**: There should a minimum of 7-days post-recovery period. All animals should be supervised daily following the animal welfare protocol. All animals that have successfully recovered from the intervention may be included in the behavioral procedures.

### Behavior shaping and optimal intensity search (OIS)

• **Behavior shaping**

Timing: 20–60 min (performance dependent).

These steps must be performed for both types of administration: self-administered (ICSS) and experimenter-administered (EAS).

**FIGURE 8:**
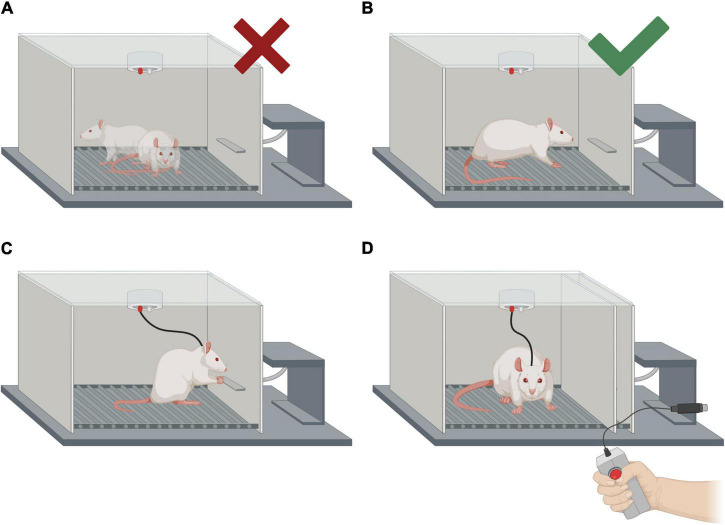
Intracranial electrical self-stimulation (ICSS) behavior shaping and representation of the two forms of treatment administration (self-administered and experimenter-administered). **(A)** Examples of the animal’s positions in which no manual stimulation should be administered. **(B)** Position of the rat in which manual stimulation should be administered in order to make the animal approach the lever. Please note that the electrode connections are not shown. **(C)** Self-administration of the treatment (ICSS). **(D)** Experimenter-administered treatment (EAS). The same apparatus is used for ICSS and EAS, but the lever must be hidden for the EAS treatment.


1. Turn on the stimulator and select a low intensity (for example, 40 μA).
2. Take the animal (a cloth can be used to restrain it) and connect the male connector to the female connector implanted in its skull.
3. Place the subject inside the Skinner box. The animal will start exploring the chamber spontaneously. Stimulation should not be administered during this initial phase of exploration (Figure 8A).
4. Press the stimulation button every time the animal approaches the wall in which the lever is located, as shown in Figure 8B.
5. The intensity can be increased gradually until the animal shows peripheral arousal (manifested by an increase in chewing demonstrated by gnawing noise, an increase in piloerection, and a general behavioral activation).
6. Once the animal can self-administer the stimulation by itself for a minute, start a stopwatch. Stop it 5 min later.
7. Write down the number of reinforcements and responses for the shaping portion.

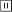
**Pause point**: After establishing ICSS behavior rats must rest for a minimum of 30 min in order to return to a basal activation level. Session OIS-1 can be done immediately after resting or 24 h after the behavior shaping session.

• **Optimal intensity search 1 (OIS-1)**

Timing: 15–30 min performance dependent.

1. Restart the counter for reinforcements and responses.
2. Reduce the intensity used during the last portion of the shaping process by 50%.
3. Start a stopwatch for 2 min. After 2 min, write down the number of responses performed at that intensity.
4. Increase intensity by 5 μA. Start a stopwatch for 2 min. After 2 min, write down the number of responses.
5. Repeat this process until the number of responses decreases, for at least two consecutive intensities. If an animal stops responding to a specific intensity for 2 min, 10 trains of stimulation may be administered manually using the button.
6. The current intensity at which the subject performs the maximum number of responses is considered its optimal intensity 1 (OI1).

**Caution!** The number of reinforcements recorded and the number of responses performed by the animal do not usually coincide. Each non-continuous current pulse lasts 0.2 or 0.3 s depending on how it is programmed. In this time interval, the rat can press the lever more than once. Responses are used to calculate the rate/unit of time, which determines the optimal intensity to be applied in the post-training treatment, while the number of reinforcements indicates how much stimulation (trains) the animal has received.


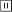
**Pause point**: A second OIS session 24 h after OIS-1 is recommended because optimal intensity usually decreases between these two sessions.

• **Optimal intensity search 2 (OIS-2)**

Timing: 15–30 min (performance dependent).

1. Follow steps in OIS-1. The current intensity in which the subject performs the maximum number of responses is considered optimal intensity 2 (OI2).
2. IMPORTANT! The mean of OI1 and OI2 is considered the optimal intensity intensity for each subject to administer the post-training treatment.

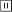
**Pause point**: The last OIS session should ideally be performed at least 72 h before the MWM training. Shorter intervals can have an anterograde effect on the performance of the task, potentially masking the effects of the post-training ICSS/EAS treatment. In this protocol, the days of the treatment administration match the behavioral training sessions, but if ICSS or EAS are designed to modulate other learning phases, these two steps should be moved in the timeline, which may result in an overlap with other behavioral procedures.

### Morris Water Maze acquisition

Timing: 15–25 min/rat performance dependent.

Before the acquisition sessions take place, one habituation session is performed in order to reduce animals’ emotional reactivity. The habituation consists in placing the animals in the pool for 60 s with no cues or platform. Once animals are removed from the pool, a cued test is performed in order to check animals’ visual acuity and motivation to find the platform. For the cued test, the platform is located in the SE quadrant. A ball with striped black and white lines is attached to the platform, ending up 20 cm above the platform surface. The cued trials consist in placing the animals in the pool to swim for a maximum of 90 s. If animals do not approach the platform during the trial, they should be manually guided to it and left there for 15 s. Habituation and cued tests were performed 3 days before the acquisition sessions.

The acquisition sessions consist of five consecutive days with six trials of 90 s each. In each trial, subjects are introduced into the pool from different cardinal points (N, E, W, S). Platform is always located in the SE quadrant. The main variable assessed was the Escape latency, which refers to the time that subjects take to find the platform. As main control variables, we measured the Time spent near walls, the swimming Speed, and the Total distance the animals swim. The time spent near walls measures thigmotaxis, which is an indicator of anxiety (Treit and Fundytus, 1988; Huang et al., 2012).


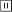
**Pause point**: Since we perform post-training stimulation, there is not an actual pause between the behavioral training and the treatment administration. The only time delay is due to the rat withdrawal from the MWM and properly drying the subject with towels before continuing with the protocol.

### Intracranial electrical stimulation treatment

Timing: 30–60 min/rat (performance dependent).

**IMPORTANT!** For all conditions, place a heating pad behind the Skinner box. Especially if animals have just performed the Morris Water Maze, in which water temperature is around 70–74°F (21–23°C).

• **Self-administration**

Self-administration treatment is depicted in Figure 8C.

1. Turn on the stimulator and select the chosen intensity (OI) for that subject.
2. Take the animal (a cloth can be used to restrain it) and connect the male connector to the female connector of the electrode implanted in its skull.
3. Place the subject inside the Skinner box.
4. Once the animal is facing the wall with the lever, press the button to administer electrical stimulation.
5. The subject should immediately start self-administering the treatment. Start a stopwatch when this happens.
6. Leave the animal self-administer 2,500 trains of stimulations.
7. Once the subject reaches 2,500 trains (reinforcements), turn off the stimulator.
8. Remove the animal and return it to its home cage.
9. Write down the total duration of the treatment and total responses.

• **Experimenter-administration**

Experimenter-administration treatment is depicted in Figure 8D.

1. Follow steps 1–3 of the self-administration protocol above.
2. A total of 2,500 trains of stimulation should be administered, aiming to deliver each train 0.5–1 s apart.
3. Remove the animal and return it to its home cage.
4. Write down the total duration of the treatment, and any relevant observations.

• **Non-stimulated animals**

1. Place the animal inside the Skinner box.
2. During ICSS shaping and optimal intensity establishment, leave animals for 20 min in the Skinner box.
3. During treatment sessions, leave them in the Skinner box for 45 min.
4. Remove the animal and place it in its homecage.

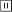
**Pause point:** This pause point depends on the timeline and the main objectives of the experiment. In this study, animals underwent a retention test 72 h after the last post-training treatment.

### Morris Water Maze retention

Timing: 3 min/rat.

Seventy-two hours after the last acquisition session, animals performed the retention test, which consisted in removing the hidden platform from the pool and placing the animal in the pool for 60 s starting from the East. Variables assessed for the retention test were analyzed for the totality of the trial duration (60 s), and the first half of the trial (30 s), given that the accuracy and level of performance could change throughout the retention test in the MWM (Blokland et al., 2004). The variables analyzed were: Percentage of time spent in the target quadrant in the first half of the trial (Q30) and its totality (Q60); Average distance to the target (DT30 and DT60), Percentage of time spent in the target annulus (Ann30 and Ann60), Time spent near walls (W30 and W60) and Wishaw’s Error (WE), which measures the time they animals spent in a 30 cm wide corridor starting at the East entry point and the platform and indicates accuracy of the trajectory.


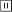
**Pause point:** Subjects were euthanized 90 min after the retention test took place.

### Tissue collection and immunohistochemical study

Timing: 2 days for IHC.

This step of the protocol aims to assess neuronal activity differences between groups after the retention session.

Ninety minutes after the retention test ended, the animals received a pentobarbital overdose (150 mg/Kg, i.p.) and were transcardially perfused with a solution of 0.1M of phosphate buffer saline (PBS), pH 7.4, followed by a solution of 4% paraformaldehyde in PBS. Brains were removed and post-fixed in 4% paraformaldehyde in PBS, then cryoprotected in 15% and 30% sucrose in PBS and stored at –80°C. Serial coronal sections (30 um) were obtained in a cryostat (Cryocut 1800, with 2020 JUNG microtome) at –20°C and stored at –80°C until immunostaining. Free-floating coronal sections were incubated with 0.3% H2O2 in Tris-buffered saline (TBS) for 30 min and in 1% Bovine Serum Albumin (BSA) in 0.3% Triton X-100 in TBS (TBS-T) for 30 min. Sections were incubated in primary antibody mouse anticFos (Santa Cruz Biotechnology Inc., Santa Cruz, CA, USA; sc-166940) at 1:500 in 0.1% BSA in TBS-T overnight at 4°C. The next day samples were incubated with biotinylated goat anti-mouse-IgG secondary antibody (Jackson Immunoresearch Inc., Ely, United Kingdom; ref.: 115-065-166) at 1:500 in 0.1% BSA in TBS-T for 60 min at room temperature and subsequently treated with Streptavidin-HRP (AKOYA Biosciences, Marlborough, MA, United States) at 1:2500 in TBS-T for 120 min, followed by treatment with 3,3′-Diaminobenzidine (DAB) solution (Vector Laboratories, Newark, NJ, United States) for 5 min. Finally, sections were mounted onto slides, dehydrated, and coverslips were placed with Pertex mounting medium (Medite GmbH, Wollenweberstraße, Germany). Negative controls without primary antibodies were included.

## Results

### Quantification and statistical analysis

**FIGURE 9:**
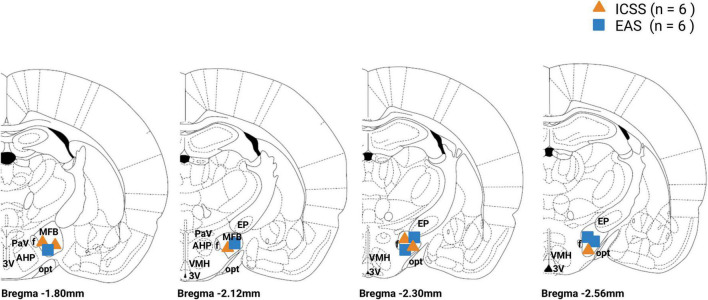
Electrode tip location for the intracranial electrical self-stimulation (ICSS) and experimenter-administered stimulation (EAS) groups. ICSS group is represented by an orange triangle and the EAS group is represented by a blue box. MFB, medial forebrain bundle; f, fornix; PAV, paraventricular hypothalamic nucleus; AHP, anterior hipotalamic area; 3V, third ventricle; opt, optic tract; EP, entopeduncular nucleus; VMH, ventromedial hypothalamus.

Statistical analyses are performed using SPSS v23 (SPSS Inc., Chicago, IL, USA). A total of 21 Wistar albino male rats (Rattus Norvegicus) were used in this experiment with a mean of age of 113 days (SD = 4.00) and a mean weight of 389.57 g (SD = 7.00) at the beginning of the experiment. Four animals were excluded from the experiment. Three subjects were excluded because they were extreme outliers in behavioral tasks (>2.0 SD points from mean in more than two acquisition sessions). One subject was excluded because of recording problems issues during behavioral tests. No animals were excluded because of electrode misplacing, as shown in Figure 9. For behavioral and molecular analysis, the total sample consisted of 17 subjects (ICSS: *n* = 6; EAS: *n* = 6; SHAM: *n* = 5). All subjects were kept in an *ad libitum* regime of food and water.

Analysis of the performance in the MWM is conducted using a 3 × 4 mixed analysis of variance (GROUP × SESSION) for the acquisition phase. An independent sample *t*-test analysis was used for the first acquisition session (S1) and the 72-h retention test. Additionally, a *t*-test was used to compare the time animals spent in the target quadrant during the retention test to the chance level (25%).

**FIGURE 10:**
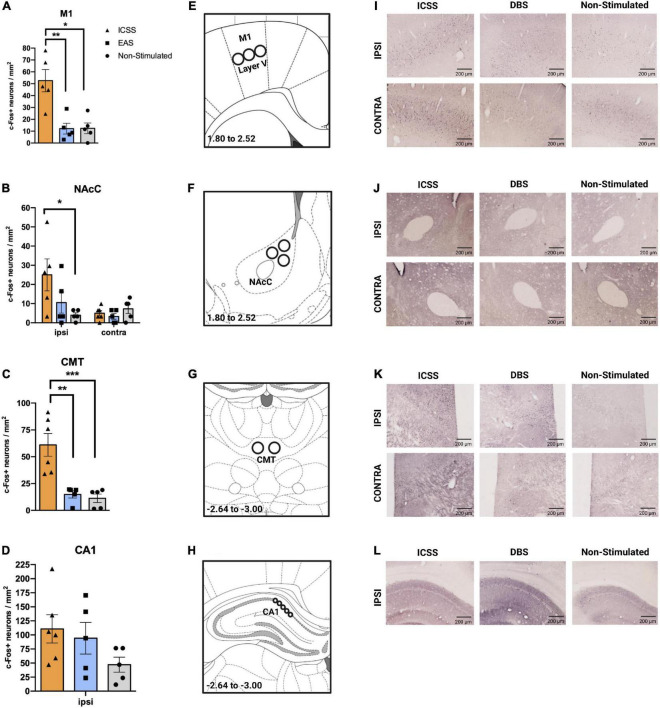
Effects of different administrations (self-administered or experimenter-administered) on c-Fos expression in the primary motor cortex, nucleus accumbens core, centromedial thalamus, and CA1. **(A–D)** c-Fos + neurons/mm^2^ (±SE) in the **(A)** primary motor cortex (M1), **(B)** NAcC, **(C)** CMT, and **(D)** CA1. The ipsilateral hemisphere corresponds to the right hemisphere, in which the electrode was implanted. Panels **(A,C)** show the mean expression of c-Fos of both hemispheres. **(E–H)** Regions of interest (ROI) for the quantification of c-Fos + neurons of the **(E)** M1, **(F)** nucleus accumbens core (NAcC), **(G)** centromedial thalamus, and **(H)** the cornus ammonis 1 (CA1), with their corresponding coordinates regarding Bregma. Images adapted from Paxinos and Watson (2007). In regions in which HEMISPHERE factor was not significant, the mean of both hemispheres is represented **(A,C)**. Significant differences are shown as *P ≤ 0.05, **P ≤ 0.01. **(I–L)** Representative images of c-Fos expression for each group and area studied taking into account the ipsilateral and contralateral hemispheres. ***P < 0.001.

To analyze c-Fos quantitative data we use a 3 × 2 mixed analysis of variance (GROUP × HEMISPHERE) for each brain area studied. When HEMISPHERE factor was not significant, the mean of the ipsilateral and contralateral hemisphere was analyzed. Microphotographs of the specific brain regions are taken with a 10 × objective lens using a BX-41 Olympus microscope attached to an Olympus DP-70 digital camera (Japan). Appropriate gray threshold and particle size are set for each area and maintained for all subjects. The image analysis software Image-J 1.43^1^ is employed to bilaterally count the number of c-Fos immunostained nuclei using regions of interest (ROIs) (see Figures 10E–H in the Section “Results”). Three histological sections are counted and averaged for all areas. In order to remove background noise, each image is digitally smoothed and subtracted from the original. See Figures 10I–L for representative immunohistochemistry outcomes.

### Optimal intensity of ICSS and responses rate evolution

**FIGURE 11:**
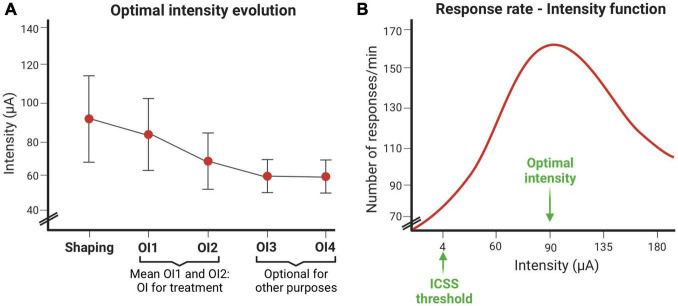
Intracranial electrical self-stimulation (ICSS) variables evolution. **(A)** Optimal intensity evolution throughout the shaping session and the optimal intensity establishment sessions. **(B)** Response rate depends on the intensity (μA) used.

The following figures depict representative optimal intensities and response rate obtained using standard ICSS parameters. Regarding OI, a decline is generally observed between OI1 and OI2 sessions, which stabilizes if more sessions are carried out. In this experiment, only OI1 and OI2 sessions were performed. However, the number of OIS sessions will depend on the purpose of the study (Figure 11A).

The response curve at different intensities usually fits an inverted-U (Figure 11B). The intensity that induces the highest response rate is called the optimal intensity. This figure also shows the intensity threshold of ICSS.

### Learning and memory outcomes

#### Acquisition

The main variable used to assess performance during the acquisition of the MWM is the escape latency, or the time subjects spend trying to find the hidden platform. No significant differences among groups in the first session (S1) were found (*P* = 0.199), which indicates that all groups began the acquisition phase at comparable levels of performance. These results allow us to rule out any effects of the previous stimulation that ICSS/EAS subjects had received before MWM training.

**FIGURE 12:**
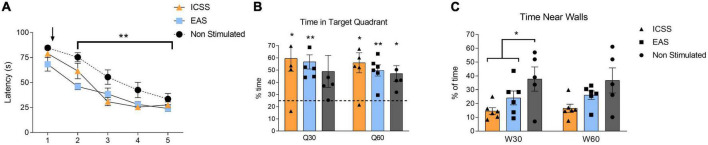
Effects of administration type (self-administered or experimenter-administered) on the acquisition and retention of a spatial task in the Morris Water Maze (MWM). **(A)** Mean escape latencies (±SE) of intracranial electrical self-stimulation (ICSS), experimenter-administered stimulation (EAS), and non-stimulated groups for the five sessions of the acquisition phase. Black arrow indicates the start of the treatment administration (self-administered, experimenter-administered, or non-stimualted). Both ICSS and EAS groups showed a significant statistically lower escape latencies compared to the non-stimulated group once the treatment had begun (S2–S5). **P ≤ 0.01. **(B)** Time spent in the target quadrant (±SE) during the retention test for the first half of the test (Q30) and the total of the test (Q60). Note that the chance level (25%) is shown in the graph as a dashed horizontal line. **(C)** Time spent near walls (±SE) during the retention test for the first half of the test (W30) and the total of the test (W60). Significant differences are shown as *P ≤ 0.05, **P ≤ 0.01.

A repeated measures ANOVA analysis (GROUP × SESSION) was used to study the escape latencies for the rest of the acquisition sessions (S2–S5). GROUP (*F*_2_,_14_ = 10.098, *P* = 0.002) and SESSION (*F*_3_,_42_ = 29.174, *P* < 0.001) factors were resulted significant, but their interaction was not significant (*F*_6_,_42_ = 1.816, *P* = 0.119). As it can be observed in Figure 12A, the evolution of the performance throughout sessions is mainly adjusted to a linear function (*F*_1_,_14_ = 77.229; *P* < 0.001), with some inflection (quadratic function: *F*_1_,_14_ = 5.431; *P* = 0.035) possibly due to the floor effect that is usually observed in the last sessions. Despite the fact that all the groups showed an improvement in the execution throughout the sessions, the two groups that received treatment showed lower escape latencies in comparison with the non-stimulated group (ICSS vs. non-stimulated: *P* = 0.003; EAS vs. non-stimulated: *P* = 0.001). Thus, both forms of administration equally facilitate the acquisition of the task. These effects start immediately after the first treatment session, and are maintained throughout the acquisition phase. No differences in control variables were observed.

#### Retention

Compared to chance level (25%) during the first half of the test (Q30), both groups that received stimulation spent more time in the target quadrant (ICSS: *t*_5_ = 3.306, *P* = 0.021; EAS: *t*_5_ = 5.469, *P* = 0.003), while the non-stimulated group did not (*t*_4_ = 1.824, *P* = 0.142). However, when we look at the whole test (Q60), all of the groups spent more time in the target quadrant compared to chance level (ICSS: *t*_5_ = 3.785, *P* = 0.013; EAS: *t*_5_ = 5.359, *P* = 0.003; non-stimulated: *t*_4_ = 3.391, *P* = 0.027), as shown in Figure 12B. Regarding the time spent near the walls during the first half of the retention test (W30), significant differences between groups were found (*F*_2_,_16_ = 4.175, *P* = 0.038). Specifically, both stimulated groups spent less time near the walls compared with the non-stimulated group (Helmert contrast: *P* = 0.021), as shown in Figure 12C. There are no differences between groups in any other variables analyzed.

### Immunohistochemistry outcomes

We measured c-Fos expression after retention at 72 h to determine neuronal activity in the primary motor cortex (M1), nucleus accumbens core (NAcC), centromedial thalamic nucleus (CMT), and hippocampal CA1 region. A mixed 3 × 2 ANOVA (GROUP × HEMISPHERE) was used.

Regarding the M1 (Figure 10A), bilateral differences were observed among groups (*F*_2_,_12_ = 6.309, *P* = 0.013). A *post hoc* (Tukey HSD) contrast showed significantly higher c-Fos expression in ICSS animals compared to the groups that did not perform the lever-press response (EAS, *P* = 0.008; non-stimulated, *P* = 0.012). Similar bilateral results were observed in the CMT (GROUP: *F*_2_,_13_ = 15.708, *P* < 0.001) (Figure 10C), where the ICSS group also exhibited more cFos + neurons than the other two groups (EAS, *P* = 0.005; non-stimulated, *P* < 0.0001).

In the NAcC (Figure 10B), differences among groups were hemisphere-dependent (GROUP × HEMISPHERE: *F*_2_,_12_ = 3.944, *P* = 0.048). In the ipsilateral hemisphere, the ICSS condition caused a greater level of c-Fos expression than the non-stimulation condition (*P* = 0.025), while the EAS group did not differ from either the ICSS or the non-stimulated groups. Due to issues in the immunolabeling of the samples, the results of the contralateral hemisphere of the CA1 region were not reliable. Based on previous work showing that ICSS causes similar bilateral activation of CA1 (Huguet et al., 2009), only ipsilateral CA1 has been included in the analysis. No differences between groups were observed (Figure 10D).

## Advantages and limitations

The main advantage of ICSS is the relative ease with which experimental animals acquire this behavior, which allows for the physiological stimulation of the BRS. In addition, it is proven to not cause dependency or addiction, so it does not involve tolerance phenomena nor resistance to extinction. Furthermore, post-mortem histological control of the location of the electrode is not necessary because the successful ICSS behavior itself is a guarantee of accurate placement. Finally, the target area is relatively large and after enough training in stereotaxic surgery, the percentage of error for accurate implantation is less than 10%. It should be noted that ICSS is considered to be innocuous and can be obtained in different species, both in male and female subjects.

One possible limitation regarding this protocol is the time of treatment administration. Depending on experimental objectives, the stimulation treatment could be administered at different times in relation to the learning task or other relevant procedures. For instance, pre-training treatment (before training) would affect the acquisition of the task, while pre-retention (before the probe test) would affect recall, and post-training (after training) would affect the consolidation process. The treatment can also be administered non-contingently to the learning process, in between phases of the training. All of these different designs have been experimentally tested for ICSS, and the post-training administration has consistently shown to be the most efficient (Segura-Torres et al., 1988; Redolar-Ripoll et al., 2002; Soriano-Mas et al., 2007). Thus, this protocol describes how to perform post-training administration of the treatment, be it ICSS or EAS.

Finally, another limitation is that the assessment of neuronal activation by c-Fos is time-sensitive (Chaudhuri et al., 2000). Thus, we are only able to link neuronal activation to the performance in the retention test, as that is the task the animals undergo 90 min before euthanasia and tissue extraction.

## Troubleshooting

**Problem 1:** The electrode implant falls down.

**Potential solution:**

(1) Avoid excessive manipulation of the electrode.
(2) Tighten the screws deeper into the skull.
(3) Before applying the acrylic cement to the skull, dry the surface completely.

**Problem 2:** An animal does not show ICSS behavior.

**Potential solution:**

(1) We can remove the animal from the Skinner box and check all connections between the stimulator, the rat electrode device, and the lever. To check that all the components of the circuit work correctly, you may use a resistor. It is advisable to have several spare cables available.
(2) Although not very common, some animals seem to receive insufficient current intensity on the first day of shaping behavior. This may be due to something that increases tissue resistance, such as a blood clot. This is usually resolved without requiring any type of intervention, so it is recommended to test the animal again at a later time.
(3) In some cases, the rat never shows ICSS behavior. For this reason, it is necessary to periodically check the stereotaxic apparatus, and be very careful in the elaboration of the electrodes. The moment of its implantation in the brain is also critical. The electrode wire should always be prevented from being accidentally bent or damaged. For example, once the hole in the skull is performed, perforate the meninges with a needle. If this step is forgotten, the electrode wire could be bent as it passes through the hard rat meninges, and this could affect the path of the electrode.

**Problem 3:** Animals display abnormal behaviors, such as stereotypic behavior, extreme peripheral arousal or even seizures during stimulation, which may hinder its ability to self-stimulate. Increased piloerection, teeth chattering, grooming and occasional ejaculation in males are normal occurrences. It is also normal for the animal to occasionally rest for short periods during the ICSS treatment.

**Potential solution:**

(1) Lower the intensity of stimulation, if possible.
(2) Closely monitor the animal’s behavior during ICSS or EAS and pay special attention to the time it takes to complete the portion of the session. If the animal continuously disengages from the ICSS behavior or if the abnormal behavior is too severe, it should not be included in the stimulation groups.
(3) Seizures: the start of a convulsive episode can be assessed by the ceasing of self-stimulation activity and slobbering. In these cases, stop the stimulation immediately. Wait a few minutes before removing the animal from the cage. General parameters of the subject should be checked over the following days. If seizures were provoked due to high intensity levels, animals may continue in the experiment, using lower intensities. Seizures do not affect performance on the learning task, but they affect the number of c-Fos + neurons, especially in the dentate gyrus, thus, depending on the molecular objectives, this should be taken into consideration.

**Problem 4:** Animals learn the behavioral task (in this case, the spatial task) faster than expected, in which case the effects of the treatment are not observable.

**Potential solution:**

(1) Make the task more difficult by reducing the number of trials per session (from 6 to 4 or to 2), or by reducing the number of the total sessions performed (Chamorro-López et al., 2015; García-Brito et al., 2018).

## Discussion

This paper shares standardized guidelines for using rewarding MFB stimulation as a treatment to improve learning and memory in rats. The materials and steps described in this protocol have been used to assess the effects of self- *vs.* experimenter-administration on the MWM performance, as well as on the expression of c-Fos after the retention test. To our knowledge, this is the first time that ICSS and EAS have been compared in a spatial memory task.

The behavioral data show that reinforcing stimulation of the MFB is an effective treatment for facilitating the acquisition of a spatial learning task in rats, regardless of whether it is self-administered or externally administered. The effects of both forms of administration are evident after the first administration of the treatment, and this effect is maintained throughout the acquisition sessions. The facilitating effect of ICSS is in agreement with previous results in the MWM (Ruiz-Medina et al., 2008; Chamorro-López et al., 2015), and other types of tasks, such as active avoidance conditioning (Segura-Torres et al., 2010). It has been shown that the potency of the effect is related to the amount of training, since non-stimulated animals are able to reach a better performance when they undergo more training (Redolar-Ripoll et al., 2002; Ruiz-Medina et al., 2008; Chamorro-López et al., 2015). This could potentially reduce the scope for action of the treatment. However, despite using a higher number of trials per session in the present study, both the ICSS and EAS-treated subjects showed an improved performance compared to the non-stimulated animals. This would be in agreement with results showing that ICSS could be even more powerful than the training itself (Redolar-Ripoll et al., 2002).

In contrast, no differences were observed between the stimulated and non-stimulated animals in the variables assessed in the retention test. Does that mean that MFB stimulation has had no long-term effects? When assessing the retention level compared to chance within each group in the first half of the test, only stimulated animals spend significantly more time in the target quadrant. This suggests that they recalled the location of the platform earlier than non-stimulated animals. Moreover, since the spatial MWM is considered to be a hippocampus-dependent memory task (Vorhees and Williams, 2014a) a study of CA1 activation was carried out. Similar c-Fos levels found in CA1 of all groups support the idea that they all end up presenting comparable retention levels, probably due to the intense training they had undergone. In fact, ICSS manages to produce long-term facilitating effects when animals receive fewer trials during acquisition (García-Brito et al., 2020). Interestingly, stimulated animals spent significantly less time near walls in the retention test compared to non-stimulated groups. This reduction in thigmotactic behavior could be related with a better retention. On the other hand, this outcome suggests a potential anxiolytic effect of the MFB stimulation.

Despite the fact that the behavioral outcome suggests equivalent therapeutic potential, the results of the c-Fos expression in M1, NAcC, and CMT, suggest possible differences between both administration forms. As expected, the ICSS group showed increased activation of the primary motor cortex, which would be explained by the persistent lever-pressing behavior required to obtain electrical stimulation (Shankaranarayana Rao et al., 1998).

Surprisingly, although both ICSS and EAS groups received rewarding stimulation, only the ICSS group showed more activation in the NAcC compared to the non-stimulated animals. This is known to be a critical region of the mesolimbic system (Baik, 2020). Since c-Fos was assessed 72 h after the last treatment administration, we cannot know whether activation of this nucleus was similar or different for both groups immediately after stimulation. Nevertheless, ICSS effects seem to be more long-lasting. Both groups received the same amount of stimulation; therefore, the most likely explanation is twofold.

## Key resources table

On the one hand, in the ICSS condition, the experimenter may readjust the intensity of stimulation based on the ICSS behavior. On the other hand, the rats in this condition are able to regulate their own level of motivation or arousal by adjusting the response rate, and usually showing obvious signs of peripheral arousal and behavioral activation (Nieh et al., 2016). Higher expression of c-Fos in the arousal-related CMT nucleus of the ICSS group could support this idea. Moreover, the ipsilaterality of the observed effect in the NAcC and bilaterality in the CMT agrees with previous results which show greater ipsilateral activation only in the areas most directly connected to the stimulated region (Arvanitogiannis et al., 1996).

In summary, both forms of administrating rewarding stimulation facilitate a hippocampus-dependent learning task, although they present differences regarding long-term activation levels in some brain regions related to arousal, and motor behavior. These results suggest that MFB is a promising target for use in DBS treatment of memory-related disorders in humans, even if the stimulation is not self-administered. Moreover, they support the idea that the results obtained using ICSS in an animal model are useful to guide clinical research.

## Data availability statement

The raw data supporting the conclusions of this article will be made available by the authors, without undue reservation.

## Ethics statement

The animal study was reviewed and approved by Departament d’Agricultura, Ramaderia, Pesca i Alimentació, Generalitat de Catalunya (DARP Ref. 7350).

## Author contributions

LV-S: investigation and writing—original draft. SG-B: investigation and writing—review and editing. LA-V: conceptualization, methodology, and writing—review and editing. EK: conceptualization, methodology, writing—review and editing, and funding acquisition. GH: conceptualization, methodology, supervision, and funding acquisition. IM-B: conceptualization. PS-T: conceptualization, writing—review and editing, project administration, and funding acquisition. All authors contributed to the article and approved the submitted version.

## Footnotes

1
http://rsb.info.nih.gov/ij/
